# Osteochondral autograft transplantation with biplanar distal tibial osteotomy for patients with concomitant large osteochondral lesion of the talus and varus ankle malalignment

**DOI:** 10.1186/s12891-016-1367-2

**Published:** 2017-01-19

**Authors:** Xingchen Li, Yuan Zhu, Yang Xu, Bibo Wang, Jinhao Liu, Xiangyang Xu

**Affiliations:** Orthopaedic Department, Ruijin Hospital, Ruijin Second Road No.197, Shanghai, China

**Keywords:** Osteochondral lesion of the talus, Supramalleolar osteotomy, Oblique medial malleolar osteotomy, Osteochondral autograft transplantation

## Abstract

**Background:**

Osteochondral lesions of the talus (OLTs) are amongst the most common foot and ankle disorders. Varus ankle malalignment causes stress concentration on medial side of the joint, resulting in OLTs and osteoarthritis. For large symptomatic OLTs (>10 mm), Osteochondral autograft transplantation is usually recommended. This article highlights biplanar distal tibial osteotomy as an approach and management for patients with concomitant large OLTs and varus ankle malalignment.

**Methods:**

From January 2012 to July 2014, 13 patients (6 male and 7 female) underwent surgery in our faculty and their average age was 55.4 (ranging from 34 to 69) years old. Oblique medial malleolar osteotomy was performed to expose the talar lesion, followed by an osteochondral autograft transplantation and distal tibial opening-wedge osteotomy. Weight-bearing X-rays were conducted and used for the measurement of radiographic parameters such as the tibial articular surface (TAS) and tibial lateral surface (TLS) angles. Ankle function of the subjects was evaluated according to the American Orthopaedic Foot and Ankle Society-Ankle and Hindfoot score (AOFAS-AH) questionnaires and Visual Analog Scale (VAS).

**Results:**

11 patients completed the follow-up over a mean period of 21.2 months. The average area of talar lesion was 135.9 mm^2^ while the average depth was 11.4 mm. The mean time for osseous union was 8.5 weeks. Donor site morbidity was not recorded in any of the cases. The mean AOFAS-AH and VAS improved from 53 to 90 points (*p* < 0.05) and 6.7 to 1.9 points (*p* < 0.05) respectively. The mean TAS angle improved from 83.1 to 90.3° (*p* < 0.05).

**Conclusions:**

Biplanar distal tibial osteotomy with the combination of osteochondral autograft transplantation could be used to address patients with concomitant large OLTs and varus ankle malalignment as this technique provides excellent visualization of the talar defect, favorable biomechanical environment for the ankle joint with high rate of good and excellent results.

## Background

Osteochondral lesions of the talus (OLTs) are commonly seen in foot and ankle injuries. According to the literature, the exact incidence rate of OLTs among population has not been well established [1]. Among the several proposed causes of OLTs [2], including trauma [3, 4], avascular necrosis [5], endocrine [6] or even genetic factors [7], ankle trauma is yet the most common etiology for OLTs [4].

Even though the probability of varus ankle deformity progressing into OLTs remains uncertain, it has been widely accepted by scholars that the prolonged exposure to eccentric loading on the medial side of the joint creates stress concentration on medial articular surface of the ankle joint [8–11] and the continuous eccentric loading may result in degenerative changes of the cartilage [10].

Patients usually present with symptoms such as medial deep ankle pain with ambulation. Some patients with long history of asymptomatic varus ankle malalignment could eventually progress into OLTs and then manifest the relative symptoms. We speculate that the main complaint and the symptoms of the patients are due to the OLTs, which could in turn be a result of from varus ankle malalignment. Hence, in quest for a more comprehensive and more effective treatment plan, it would be more convenient to simultaneously address to OLTs and varus ankle malalignment in patients with both.

The choice of the management strategy for OLTs depends on factors such as clinical manifestations, lesion size and location and surgeons’ preference [12]. For symptomatic lesions smaller than 10 mm, conservative or debridement and microfracture under arthroscopy is indicated while for symptomatic lesions greater than 10 mm or secondary lesions, osteochondral autograft transplantation, autologous chondrocyte implantation (ACI) or allograft transplantation might be preferred [2, 13, 14]. Medial malleolar osteotomy is often recommended for the full access to the talar lesions, particularly in lesions involving posteromedial aspect of the talar dome [15]. The main polemic is which approach to choose if the patient has concomitant varus ankle malalignment. Hence, the purpose of this article is to highlight the feasibility of biplanar distal tibial osteotomy as an approach and management for patients with large OLTs and concomitant varus ankle malalignment. The proposed technique is a combination of oblique medial malleolar osteotomy and distal tibial opening-wedge osteotomy: the oblique medial malleolar osteotomy provides excellent visualization for talar defect; the OLTs are managed with osteochondral autograft transplantation while the varus ankle malalignment is corrected by distal tibial osteotomy. The clinical outcome has been further evaluated in this study.

## Methods

Over the period from January 2012 and July 2014, 13 patients who were diagnosed with OLTs and concomitant with varus ankle malalignment underwent biplanar distal tibial osteotomy and osteochondral autograft transplantation in our foot and ankle department (Table 1).

**Table 1:** Patient Information

Case	Sex	Age	Follow up (months)	Trauma	Side	Location	Area	OLTs	Satisfaction	AOFAS	VAS
1	F	66	30	Yes	R	4,7	12×7×9	Ф6mm×2 plugs	good	53–90	6–2
2	F	58	36	No	R	4	10×10×12	Ф8mm×1plug +cancellous bone graft	fair	49–81	7–3
3	F	69	15	Yes	L	4,7	14×12×10	Ф8mm×1plug + cancellous bone graft	good	62–90	5–1
4	F	52	17	No	R	4	8×12×12	Ф8mm×1plug + cancellous bone graft	good	60–90	5–1
5	F	61	20	Yes	R	4,7	15×7×8	Ф8mm×1plug + cancellous bone graft	excellent	47–100	9–2
6	M	63	15	No	L	4,7	16×12×10	Ф8mm×2 plugs	fair	74–80	9–5
7	M	52	12	No	L	1,4,7	15×16×14	Ф8mm×2 plugs	excellent	56–100	6–1
8	F	54	24	Yes	L	4,7	14×12×13	Ф6mm×2 plugs + cancellous bone graft	good	38–90	7–2
9	F	63	32	No	R	4,7	16×8×12	Ф8mm×2 plugs	excellent	56–100	6–1
10	M	48	N/A	No	L	4	10×13×10	Ф8mm×1plug + cancellous bone graft	N/A	–	–
11	M	53	18	Yes	L	4,7	12×8×16	Ф8mm×1plug + cancellous bone graft	excellent	45–90	8–1
12	M	34	14	Yes	L	4,7	15×10×12	Ф8mm×2 plugs	good	39–84	6–2
13	M	47	N/A	No	R	4	8×12×10	Ф8mm×1 plug+ cancellous bone graft	N/A	–	–

Preoperative weight-bearing stress radiographs were obtained using a TELOS stress device (TELOS, METAX, Germany) as described by Karlsson [16]. The pressure load was 150 N. The indications for the reconstruction of lateral ligaments of the ankle joint were more than two episodes of recurrent ankle sprains within half of a year and severe ankle instability (more than 10° of talar tilt or more than 10 mm of anterior displacement). The anatomical reconstruction of the lateral ligaments of the ankle joints was performed using a minimally invasive approach [17, 18].

**Fig. 1:**
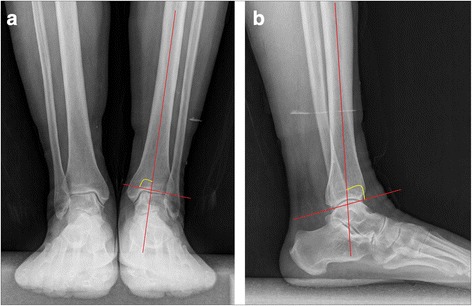
Radiographic measurements. **a** TAS angle. **b** TLS angle

Weight-bearing X-ray and Saltzman’s hindfoot alignment view [19] of the ankle joints were used for the evaluation of bony deformity. Patients who had deformities at the level of tibial shaft or on the lower extremity were excluded. The tibial anterior surface and tibial lateral surface angles of the subjects were measured [20, 21] (Fig. 1). In order to determine the location size and depth of the talar defect, MRI and CT scans were also conducted. A nine-zone grid system on the talar dome was used to describe the location of lesion. The articular surface of the talar dome was divided equally into nine zones equal in area and axial plane, with zones 1,4 and 7 positioned on the medial talus, zones 3,6 and 9 positioned on the lateral talus zone, zones 1,2 and 3 positioned on the anterior talus and zones 7,8 and 9 positioned on the posterior talus [22].

**Fig. 2:**
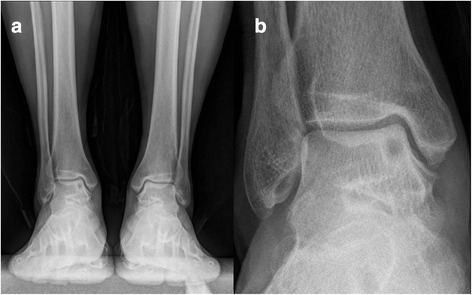
A 63 year old female with concomitant large OLTs and varus ankle malalignment. **a** Weight-bearing AP view showed a lucent area on the medial side of left talus. **b** Magnified view of the talar defect

**Fig. 3:**
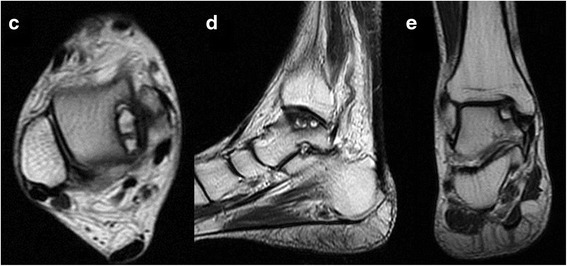
A 63 year old female with concomitant large OLTs and varus ankle malalignment. **c**-**e** A large subchondral cyst was found with high signals at the medial talar shoulder on T2-weighted images

**Fig. 4:**
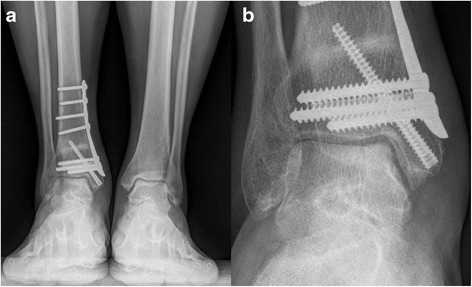
**a** Weight-bearing AP view of the same patient at 8 month after operation. **b** Magnified view of the talar defect

The indications for biplanar distal tibial osteotomy are: (1) large OLTs (>10 mm); (2) defect is located in zones 4 and 7. (3) Varus ankle malalignment, typically with more than 10° of distal tibial angulation (Figs. 2, 3 and 4).

### Surgical technique

Patient was positioned supine with a pneumatic tourniquet on the upper thigh. After general or spinal anesthesia, a 10 cm longitudinal incision was made over the medial malleolus. The great saphenous vein was very carefully located and protected. Oblique medial malleolar osteotomy was performed approximately 5 cm above the tip of medial malleolus. The distal fragment was flipped downwards to allow further visualization of the talar lesion. After the insertion of a 6 mm or 8 mm recipient tube harvester (Osteochondral Autograft Transfer System, Arthrex, USA), the unhealthy osteochondral plug was taken out. The wall and base of the lesion were abraded and curetted down to viable subchondral bone. Then the articular cartilage was reconstructed by using cylindrical autologous osteochondral plugs taken from the ipsilateral knee joint. Cannulated screws were introduced to stabilize the distal tibia fragment after reduction.

**Fig. 5:**
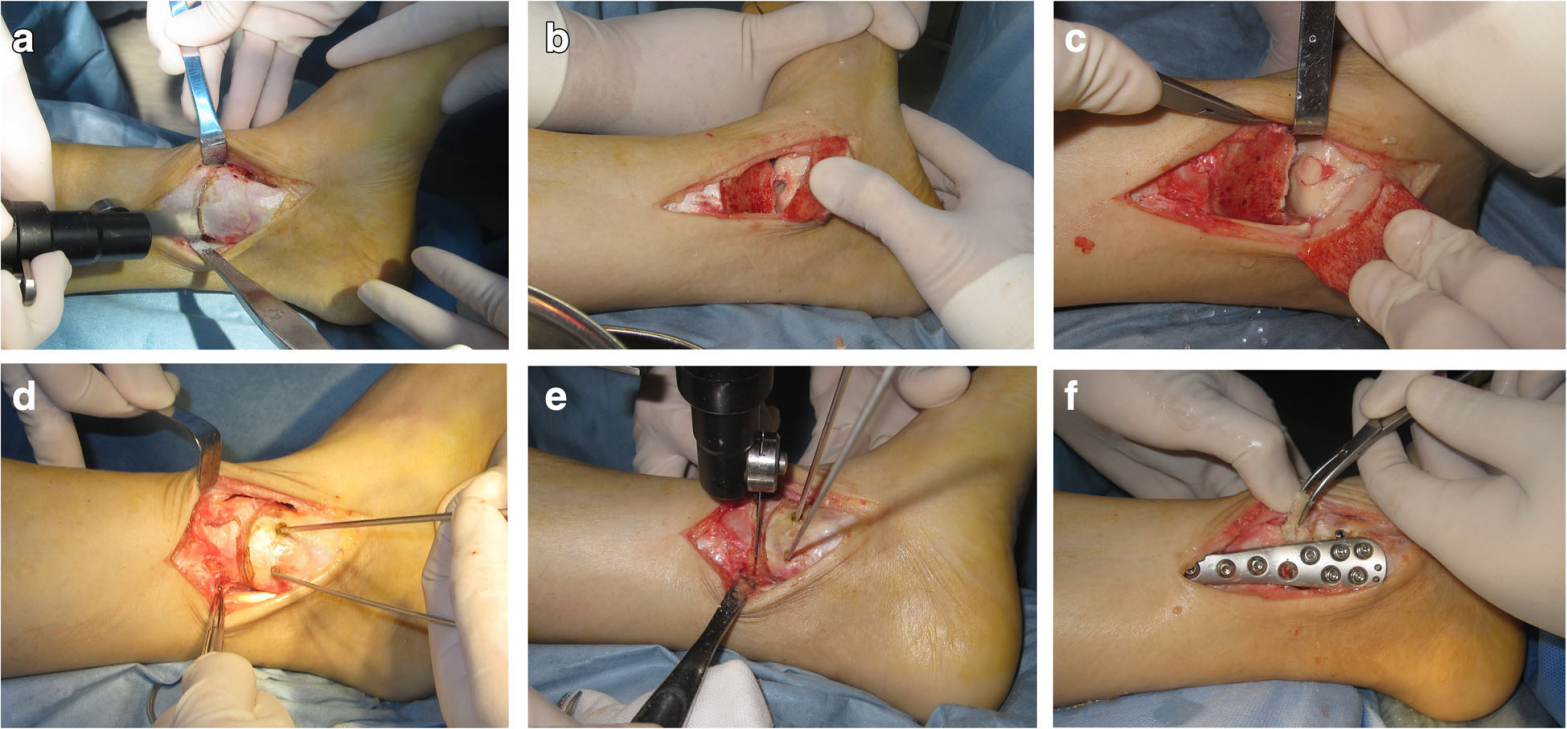
Biplanar distal tibial osteotomy and osteochondral autograft transplantation. **a** The distal tibia was exposed, medial malleolar osteotomy was performed about 5 cm above the tip of medial malleolus. **b** The distal fragment was flipped downward for direct visualization. The wall and base of the lesion were abraded and curetted down to viable subchondral bone. **c** Cylindrical autografts taken from the ipsilateral knee joint was inserted into the defect to resurface the articular cartilage. **d** The fragment was stabilized with two cannulated screws parallel to distal tibial plafond. **e** The distal tibial osteotomy was performed about 5 cm above the medial malleolar tip, which was almost at the same level of medial malleolar osteotomy. **f** The osteotomy was stabilized with plates and screws

The horizontal distal tibial osteotomy was performed about 5 cm above the medial malloelus, which is almost at the same level of medial malleolar osteotomy. The cut was opened with a Hintermann spreader to the desired amount. Special attention was paid to keep the lateral cortex intact so that it could remarkably increase the stability of the osteotomy while acting as a fulcrum for the opening-wedge. The tricortical allograft was used as bone graft (Osteorad Ltd, Shanxi, China). Eventually, the osteotomy was stabilized with plates and screws (Fig. 5) [23–25].

### Postoperative care

The ankle and knee joint were wrapped in bandage for 2 weeks. The patients were allowed to begin passive range of motion of the ankle and knee joint at 2 weeks after operation. The patient wore removable walking boots, and started partial weight-bearing at 4^th^ week post-operation. At 6^th^ week, full weight-bearing began as X-ray showed the evidence of bone healing, that is callus formation at the site of supramalleolar osteotomy.

### Statistical methods

All patients were evaluated according to both clinical and radiographic standards. AOFAS-AH questionnaires and VAS were used to evaluate the patients before surgery and at the time of last follow-up. Patient satisfaction level was graded as excellent, good, fair or poor. Radiographic parameters included the TAS angle and TLS angle.

SAS software (version 8.02, SAS Institute, USA) was used for statistical analysis. Paired student t-test was conducted for the evaluation of changes in AOFAS-AH score, VAS and radiographic parameters. A P value less than 0.05 was considered to be statistically significant.

## Results

Among the 13 patients who were involved in this study, there were 6 male and 7 female with an average age of 55 (range, 34 to 69) years old at the time of surgery. Among the 13 cases, there were 7 cases of left foot lesion and 6 cases of right foot. 1 patient had a history of tibial shaft fracture while 5 patients had history of ankle sprain; the remaining 7 did not have history of trauma.

11 out of 13 patients completed the follow-up. We lost contact with one of the patients while another patient refused to continue the follow up. The mean final follow-up time was 21.2 (range, 12 to 36) months. The average area of the talar lesion was 135.9 mm^2^ while the average depth of the lesion was 11.4 mm. All patients had talar defect involving zones 4 or 7. The mean AOFAS-AH score increased from 53 to 90 points (*t* = 9.3, *p* < 0.05). The AOFAS-AH subscale for pain improved from 20.9 (range, 20 to 30) preoperatively to 32.7 (range, 30 to 40) postoperatively. The subscale for function improved from 31.7 (range, 18 to 44) to 47.7 (range, 40 to 50) postoperatively. The alignment subscale improved from 0 preoperatively to 10 postoperatively. The mean VAS improved from 6.7 to 1.9 points (*t* = −14, *p* < 0.05).

**Fig. 6:**
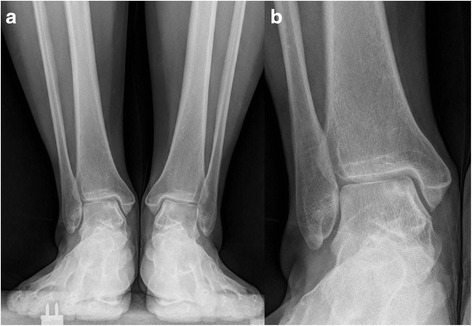
**a** A 58 year old female with concomitant large OLTs and varus ankle malalignment. **b** Magnified view of the talar defect

**Fig. 7:**
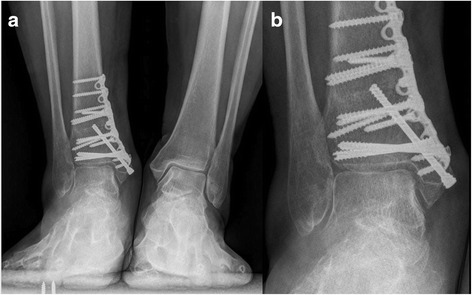
**a** Weight-bearing AP view of the same patient at 3 month after operation. **b** Magnified view of the talar defect

**Fig. 8:**
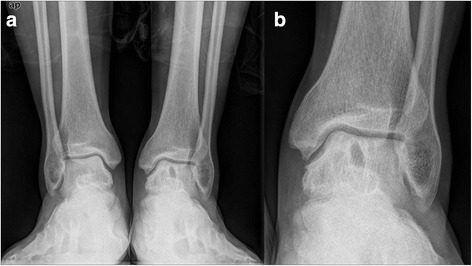
**a** A 52 year old male with concomitant large OLTs and varus ankle malalignment. **b** Magnified view of the talar defect

**Fig. 9:**
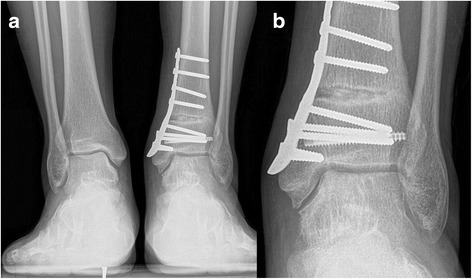
**a** Weight-bearing AP view of the same patient at 3 month after operation. **b** Magnified view of the talar defect

Radiographically, the TAS angle improved from a mean of 83.1 to 90.3° (*t* = 5.4, *p* < 0.05). The TLS angle showed no statistically significant change with 78.3 ± 3.6° pre-operatively and 79.6 ± 3.3° post-operatively (*t* = 2.2, p > 0.05). Post-operative X-rays were obtained for all patients. Radiographic osseous union time averaged 8.5 weeks, there was no malunion for oblique medial malleolar osteotomy and supramalleolar osteotomy. All patients showed a good incorporation of the graft with the surrounding cartilage and subchondral bone. No subsidence or elevation of the graft was identified. No patient reported donor site morbidity at the time of last follow-up (Figs. 6, 7, 8 and 9).

**Fig. 10:**
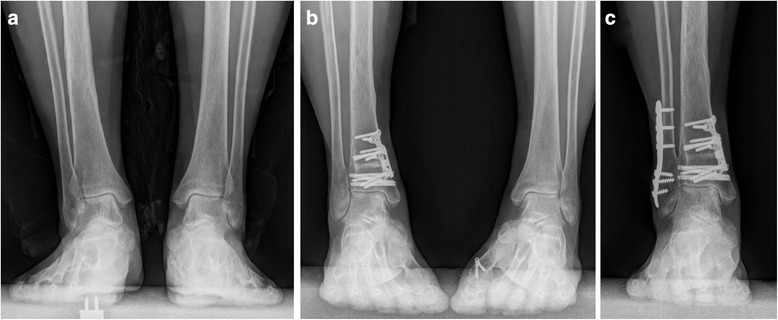
A 66-year-old female with OLTs concomitant with distal tibial varus. Biplanar distal tibial osteotomy and osteochondral autograft transplantation were performed. The patient was re-hospitalized because of subfibular impingement and underwent fibular osteotomy and ankle joint clearance 14 months after initial operation. The patient was satisfied and pain free at the time of last follow up. **a** Weight bearing AP view of the ankle joint pre-operation. **b** 1 year after the initial operation. **c** 1 year after the second operation

Two patients complained of pain in the lateral aspect of the calcaneus, but the medial deep ankle pain relieved after operation. The TAS angle improved from 84.5 to 92.8° for one patient, we believe subfibular impingement causes the pain as a result of overcorrection. One of them was re-hospitalized and underwent fibular osteotomy and ankle joint clearance 14 months after initial surgery. And she was satisfied and pain free at the time of last follow-up. The other patient had mild symptom, she refused the operation and was still satisfied with the primary operation (Fig. 10).

At the time of last follow-up, subjective patient satisfaction (excellent, good, fair, poor) revealed that 9 (81.8%) of 11 patients rated the result as good or excellent and the other 2 (18.2%) rated as fair. The AOFAS-AH score for patients who rated as fair is 81 and 80 points respectively. They complained of moderate pain and decreased range of motion of the ankle joint. No degenerative change was identified in these patients and they were treated with medication and rehabilitation.

## Discussion

OLTs are commonly seen in foot and ankle injuries. Even though the diagnosis can be easily made by combining patient history, physical examination and imaging, the management of OLTs however still remains a challenge for orthopaedic surgeons [2, 14]. Trauma is widely accepted as the most common etiology of OLTs. In lateral lesions, 98% of cases have a history of trauma; and in medial lesions, the incidence is 70% [5]. The lateral lesion is shallow, indicating a shear mechanism of injury, while the medial lesion is deep and cup-shaped, indicating a mechanism of torsional impaction and axial loading [13, 26]. In spite of the prevalence of different factors related to nontraumatic etiology, eccentric loading plays a role in OLTs [13, 27, 28]. Continuous eccentric loading on the medial side of the ankle joint can result in degenerative change of cartilage [10]. Once the subchondral bone plate is broken, ankle fluid can be forced into the subchondral bone. Cyclic loading of the joint can eventually lead to subchondral cyst, stimulating nerve endings in the subchondral bone and cause symptoms [13, 26].

Supramalleolar osteotomy is designed to correct the medial displacement of the load line, laterally distributing the medial concentration of stress within the ankle joint [21], providing favorable biomechanical environment for cartilage repair [21, 29, 30]. Numerous publications have proved its efficacy in managing early stage ankle osteoarthritis [9, 20, 23–25, 31–35]. Though none of the patients in this study has concomitant ankle osteoarthritis, the goal of the osteotomy is to correct the malalignment of the distal tibia and redistribute stress within the ankle joint.

What provides excellent clinical results? In review of the literature, osteochondral autograft transplantation provides encouraging good and excellent clinical results. Hangody et al. [36] reported 92% good to excellent outcomes with 93 patients at mean follow up of 9.6 years. Gautier at al [37] reported 91% overall satisfaction rate with 9 patients at mean follow up of 2 years. Valderrabano at al [38] reported 92% of good and excellent results with 12 patients at mean follow up of 6 years. Zengerink et al. [13] reported a success rate of 87%. Although there are numerous studies reported high good and excellent rates, none of them reported the alignment of the affected ankle joint. In 2012, Easley et al [8] reported 2 cases who had concomitant varus ankle deformity and OLTs, and treated with both alignment correction and cartilage reconstruction. However, there has been very less reports on OLTs with concomitant varus ankle malalignment in literature. Well-designed clinical studies and biomechanical studies may be needed to fully understand these two problems.

Though most of the lesions can be managed successfully under arthroscopy [39]. In case of large lesion localized on the posteromedial talar dome, medial malleolar osteotomy should be indicated [15, 40]. Different approaches for medial malleolar osteotomy have been described in literature, transverse osteotomy [41], oblique osteotomy [15], crescent osteotomy [42], inverted V osteotomy [43], step cut osteotomy [40, 44]. The modifications of the procedure have improved joint visualization and eliminated complications, such as malunion of the medial malleolus and iatrogenic talar dome damage. Oblique medial malleolar osteotomy is preferred in these patients because this approach is technically simple and allows excellent visualization of the talar defects. However, it has the potential for complications, such as proximal migration of the distal fragment, rotational malunion and iatrogenic talar dome damage. The osteotomy is predrilled to eliminate proximal migration and rotational displacement after fixation. Stabilizing the fragment with screws parallel to distal tibial plafond and lag screws perpendicular to the osteotomy can also prevent proximal migration [40].

In this study, two patients complain of pain in the lateral aspect of the calcaneus after operation. The pain can be induced by weightbearing and it was located between the tip of the fibular and the lateral of the calcaneus. Subfibular impingement is the source of pain in lateral aspect of hindfoot in severe adult flatfoot deformity as the result of valgus deformity of the calcaneus. Cyst formation or sclerosis in this region on plain radiographs or CT scans should create suspicion of impingement [45]. Medial opening wedge osteotomy of the distal tibia has the potential to create an overcorrection and cause subfibular impingment. Careful pre-operative planning is essential to prevent this complication.

Our study has several limitations. The first is its retrospective nature, a limited patient population and follow-up. 11 of the 13 patients completed the follow up with the mean follow-up of 21.2 months. Another limitation is that MRI or CT scans are not obtained postoperatively, though X-ray is obtained postoperatively for all patients, it is less sensitive and has limitations to evaluate cartilage and subchondral bone. Additional retrospective and prospective studies and longer term studies on larger patient population will be needed to ultimately determine the efficacy of this technique.

## Conclusion

﻿Biplanar distal tibial osteotomy with the combination of osteochondral autograft transplantation could be used to address patients with concomitant large OLTs and varus ankle malalignment. The medial malleolar osteotomy allowed excellent visualization of the talar defect, while supramalleolar osteotomy provided favorable biomechanical environment for the ankle joint. This technique achieved high good and excellent results according to our early follow up.

## Abbreviations

ACI: Autologous chondrocyte implantation
AOFAS-AH: American orthopaedic foot and ankle society-ankle and hindfoot
CORA: The center of rotation and angulation
OLTs: Osteochondral lesion of the talus
TAS: Tibial articular surface
TLS: Tibial lateral surface
VAS: Visual analog scale
